# Stress-Driven Accelerated Evolution and Ecological Network Reconfiguration in Extremophilic Microbial Communities

**DOI:** 10.3390/biology15110841

**Published:** 2026-05-28

**Authors:** Han Zhu, Liang Zhang, Zhao Hao, Enyong Chen, Yanhong Wang, Huaiming Jin, Yonghong Zhou

**Affiliations:** 1 Key Laboratory of Biodiversity and Environment on the Qinghai-Tibetan Plateau, Ministry of Education, Xizang University, Lhasa 850000, China; 2 School of Ecology and Environment, Xizang University, Lhasa 850000, China

**Keywords:** extreme environments, extremophiles, stress-driven evolution, horizontal gene transfer, ecological networks

## Abstract

Persistently high levels of abiotic stress define extreme environments. Even for adapted extremophiles, we argue this stress remains a continuous physiological challenge, necessitating energetically costly homeostasis. Crucially, this persistent pressure drives a self-reinforcing feedback loop across biological scales: it accelerates genomic evolution and concurrently reshapes ecological network architecture. Genomic innovations provide new traits for network reconfiguration, while the restructured network acts as a selective filter guiding subsequent evolution. This loop underpins extreme ecosystem resilience—the capacity for stress-induced adaptive restructuring. We synthesize mechanisms of this stress-adaptation interplay, propose testable hypotheses and outline experimental evolution approaches to validate this predictive framework for microbial responses to global change.

## Simple Summary

Life is found almost everywhere on Earth, including places with extreme heat, salt, or pressure. These extreme environments are home to unique microbes, known as extremophiles, that are specially adapted to these harsh conditions. But does living in such a place still feel “stressful” to them? Evidence affirms that stress remains a continuous pressure, necessitating a high-maintenance homeostasis sustained by constant energy investment. This persistent stress acts as a powerful engine for change. First, it speeds up the rate at which the microbes’ genetic code changes and is shared between different organisms, leading to new abilities faster than normal. Second, this stress reshapes how these microbes interact with each other, effectively rebuilding the social and competitive networks within their community. Importantly, these two processes fuel each other. Genetic changes provide the tools for new types of interactions, and the new community structure, in turn, influences which genetic changes are most useful in the future. Understanding this cycle is crucial. It explains how life can thrive at its very limits and helps us predict how microscopic ecosystems might respond to our planet’s rapid environmental changes. The lessons learned from life on the edge could also inspire new innovations in biotechnology.

## 1. Introduction

Extreme environments are those in which one or more physicochemical conditions consistently approach or exceed the tolerance limits of most life forms [1]. Representative examples include deep-sea hydrothermal vents [2], saline lakes [3], and the hadal zones of the ocean [4]. The microorganisms that inhabit these niches, collectively known as extremophiles, have evolved unique survival strategies over vast evolutionary timescales, thereby continually redefining the limits of biological tolerance. These extreme environments have revealed numerous deeply branching and novel microbial taxa, thus profoundly expanding our understanding of life’s diversity [5,6,7].

For non-extremophiles, extreme conditions like high temperature or salinity are lethal. Extremophiles, by contrast, possess specialized adaptations [8,9,10,11,12,13,14], which might make it appear that the very conditions lethal to others have become largely negligible for extremophiles. However, the extreme pressures are not static filters but persistent, dynamic drivers of change. Even for these specialists, the stress remains a continuous physiological challenge. For instance, thermophiles must constitutively manage protein unfolding at high metabolic cost [15,16]; acidophiles continuously expel H^+^ at a significantly increased ATP demand [17]. Such persistent homeostatic demands require constant energy investment. Thus, adaptation in extremophiles is not the absence of stress but its perpetual, energy-intensive management.

At the population and community levels, this continuous physiological management transforms abiotic stress into a persistent driver of a dual process—accelerated microbial evolution and ecological network reconfiguration. These two levels are interconnected and mutually reinforcing, jointly shaping ecosystem stability, function, and evolutionary trajectory. Sustained stress elevates evolutionary rates by increasing genetic variation, modulating horizontal gene transfer (HGT), and imposing strong directional selection [18,19,20]; the resulting adaptive changes then provide the genetic basis for novel interactions, restructuring the ecological network and determining its resilience. This reciprocal dynamic constitutes a feedback cycle that operates across molecular and community scales.

Here, we systematically dissect this feedback cycle. First, we detail the specific molecular mechanisms—from error-prone polymerases to chaperone systems—through which stress directly accelerates genetic variation. Second, we link these genomic innovations to quantifiable changes in network topology. Finally, we integrate these scales to propose testable hypotheses regarding the resilience and evolutionary trajectory of life under extreme conditions.

## 2. The Dynamic and Compound Nature of Environmental Stressors

In extreme environments, the ecological and evolutionary significance of abiotic stressors lies not merely in the instantaneous challenge they pose to organisms (Table 1). More critically, it lies in their multidimensional complexity, which includes how stressors fluctuate over time and how multiple stressors interact with one another in many cases. Temporal dynamics, including periodic or stochastic fluctuations, are inherent even to environments dominated by a single stressor. Superimposed on the temporal dimension, the simultaneous or sequential occurrence of multiple stressors introduces additional coupling effects, such as synergy or antagonism, that cannot be deduced from any single stressor considered in isolation. Together, these attributes form a sophisticated “stress system” that transcends simple tolerance models. Adaptation to extreme environments, therefore, is fundamentally an evolutionary response to this dynamic and interactive network of abiotic pressures [21,22,23,24,25,26].

Understanding the evolutionary and ecological implications of this complexity requires distinguishing two qualitatively distinct types of extreme environments. Stable extreme environments—such as deep subsurface brines, abyssal hadal trenches, and highly acidic geothermal pools—maintain physicochemical parameters near-constant and beyond the biokinetic range of most life. In these settings, selection is unidirectional. Despite high absolute stress, the maintenance cost is predictable. Microbial communities accordingly tend to evolve into streamlined, low diversity, and highly specialized consortia [27]. Evolution is predominantly purifying, pruning non-essential functions. In contrast, fluctuating extreme environments exhibit temporal oscillations, as seen in intertidal salt marshes, alpine permafrost, and desert biological soil crusts. The relentless shifting of stress boundaries keeps DNA repair systems alert, sustains competence for horizontal gene transfer, and forces continuous network reorganization [28]. This distinction clarifies why the highest rates of evolutionary innovation often emerge not from the hottest or saltiest extremes, but from interfaces and transition zones where stress is both high and variable.

This dichotomy manifests clearly when comparing organisms that inhabit different positions along the stability spectrum. *Thermoacidophilic* archaea of the order *Sulfolobales*, which dominate stable, sulfur-rich geothermal pools, maintain a constitutively low membrane permeability by incorporating tetraether lipids with cyclopentane rings, a structural adaptation that is energetically expensive but permanently required [29,30]. Halophilic archaea inhabiting seasonally fluctuating salt lakes, by contrast, dynamically remodel their membrane lipid composition—shifting between glycerol diether and bilayer-forming lipids—in response to changing salinity—a flexible but fast-acting strategy that conserves energy during periods of moderate osmotic pressure [31]. These contrasting “hard-wired” versus “responsive” investments recur across the domains of life and across biological scales, from membrane architecture to DNA repair systems.

The contrasting strategies of constitutive versus inducible investment in proteostasis provide a molecular illustration of this dichotomy. In hyperthermophilic archaea inhabiting stable extreme environments, the group I chaperonin (Thermosome) is constitutively overexpressed to massive levels, representing a fixed metabolic cost suited to relentless, predictable stress [32]. In contrast, organisms in fluctuating extreme environments rely on the DnaK/DnaJ/GrpE (Hsp70) system, which is strongly inducible via transcriptional regulators such as HrcA [33]. This “hard-wired” versus “flexible” divide extends beyond chaperones to osmolytes, DNA repair systems, and membrane remodeling pathways, forming a consistent adaptive logic that distinguishes microbial strategies across the extremophilic spectrum [34]. Yet the adaptive logic itself is shaped fundamentally by the temporal structure of stress—a dimension that warrants closer examination.

That temporal dimension is most pronounced in fluctuating environments, where key physicochemical parameters exhibit pronounced periodic or pulsed fluctuations [35,36,37,38,39,40,41]. This variability presents a greater challenge than stable extremes, as it demands physiological plasticity and metabolic resilience to cope with continually shifting stress intensities [42,43,44]. For instance, in arid and semi-arid ecosystems, diurnal extremes combine high daytime temperature, low humidity, and intense radiation, causing severe water deficit and photo-oxidative damage; nighttime temperature drops, however, may permit transient rehydration via dew formation [45]. Consequently, biological activity is compressed into narrow temporal windows, necessitating precise synchronization of water-use strategies, metabolic rhythms, and repair mechanisms with these highly predictable stress-relaxation cycles [46,47,48,49,50]. In hypersaline habitats such as salt marshes and seasonal salt lakes, salinity oscillates widely due to evaporation, precipitation, and tidal influence, favoring the evolution of sensitive osmosensing pathways and rapid regulatory networks for the synthesis and degradation of compatible solutes to maintain turgor and ion homeostasis [14,51,52]. In polar and alpine environments, macro-seasonal fluctuations dominate life-history strategies: prolonged polar nights or winters bring persistent cold, freezing, and resource scarcity, whereas brief growing seasons bring ice melt, moderate temperatures, and continuous light [53]. Organisms must therefore employ strategies such as deep dormancy, freeze tolerance, and highly compressed life cycles to align growth and reproduction with these short favorable periods [54,55,56,57]. In summary, the persistence and evolution of life in extreme environments reflect a comprehensive adaptation to a multivariate stress field that is interactive in factor space and dynamic in time. Temporal fluctuations shape the rhythms of physiological elasticity and behavioral plasticity, while multidimensional complexity drives the evolution of systemic and integrated adaptive strategies.

The co-occurrence and interaction of multiple stressors create multidimensional complexity [58,59]. These combined effects are often nonlinear, manifesting as synergistic enhancement or, occasionally, antagonism—where the total impact exceeds or falls short of the sum of individual stresses [60,61,62,63,64,65,66]. Synergistic effects substantially increase the difficulty of adaptation. A classic example is the salt–drought combination: this co-stress not only jointly lowers environmental water potential, causing severe osmotic stress, but also intertwines ion-specific toxicity (such as Na^+^ competition with K^+^ uptake), ionic imbalance, and dehydration-induced metabolic disruption, simultaneously attacking multiple cellular functions [67,68,69,70,71,72,73]. Another example is deep sea environment, where the interaction between low temperature and high pressure shows complexity in this setting: both generally act synergistically to reduce membrane fluidity, challenging membrane protein function and transport; however, the physical effect of pressure on water structure can partly inhibit ice-crystal formation and growth, thereby offering some antagonism to freezing injury [74,75,76,77,78,79,80]. Thus, under combined stress, an organism’s overall resilience depends not only on its tolerance to specific primary stressors but also on its integrated antioxidant defenses, damage-repair capacity, and homeostatic networks that maintain energy metabolism and redox balance [81,82]. Crucially, oxidative stress frequently serves as a common secondary pathway converging from diverse primary stressors [83,84,85,86,87]. The thiol-disulfide redox buffering systems—thioredoxins, glutaredoxins, and peroxiredoxins—are central to managing this challenge and intersect directly with gene regulation through redox-sensitive transcription factors such as OxyR and SoxRS [88,89]. In anaerobic extreme environments, reductive stress—defined by an excess of reducing equivalents (elevated NADH/NAD^+^ and NADPH/NADP^+^ ratios)—poses a challenge as fundamental as oxidative stress, yet it has received far less attention. When electron acceptors are scarce, over-reduced electron carriers accumulate, inhibiting key catabolic dehydrogenases and disrupting the cellular redox poise required for biosynthetic reactions. To counter this, strict anaerobes deploy specialized ferredoxin-thioredoxin reductases (FTRs) that channel excess reducing power from ferredoxin to thioredoxin, thereby modulating the redox state of downstream target proteins [90,91]. This system has been biochemically characterized in methanogenic archaea such as *Methanosarcina acetivorans*, where a recently described group 4 FTR-like enzyme uses unique [4Fe–4S] clusters to regulate catalytic activity in response to cellular redox status, directly linking environmental electron availability to the transcriptional and post-translational reprogramming of metabolism [90]. In stable anaerobic extremes such as deep subsurface brines, where electron acceptor limitation is chronic and predictable, reductive stress responses are constitutively engaged; in fluctuating anaerobic interfaces such as intertidal sediments, where redox conditions oscillate with tidal cycles, these systems must be rapidly inducible to match the shifting availability of terminal electron acceptors.

## 3. Stress-Driven Acceleration of Genomic Innovation

### 3.1. Stress-Induced Elevation of Mutation Rates

**Figure 1:**
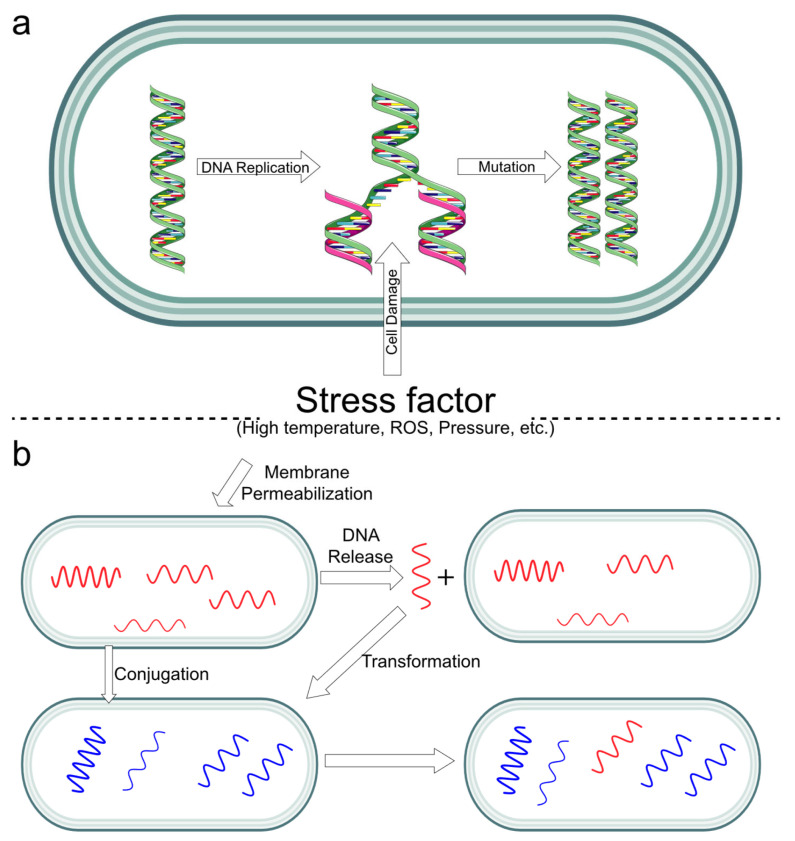
The impact of extreme environmental factors on microbial evolution. (**a**) Extreme environmental factors act as a driver for DNA mutations in microorganisms. Different colors on the parental strand represent the four canonical bases. Stress factor induces base misincorporation during replication, yielding mutated daughter strands with altered base pair composition.; (**b**) Extreme environmental factors influence horizontal gene transfer in microorganisms. Red DNA indicates genetic material originating from the donor cell; blue DNA indicates genetic material originating from the recipient cell. DNA release, transformation, and conjugation collectively introduce donor DNA into the recipient, producing a new cell that harbors both recipient (blue) and donor (red) genetic information.

Extreme stress accelerates microbial evolution by directly increasing genetic diversity through elevated mutation rates [92,93]. This occurs via a molecular cascade: stressors increase DNA damage load and complexity, overwhelm high-fidelity repair systems, and activate error-prone damage tolerance pathways (Figure 1a) [94,95,96].

Multiple stressors act collectively to raise the mutation rate and diversity of DNA lesions [95,97,98,99]. For instance, high temperature causes structural damage and interferes with DNA replication process [100,101]; ionizing radiation and ultraviolet light directly induce strand breaks and cyclobutane pyrimidine dimers [102,103]; oxidative stress—triggered by heat, radiation, high salinity, or heavy metals—leads to a surge in intracellular reactive oxygen species (ROS), resulting in widespread oxidative base modifications [104,105]. If unrepaired, these lesions can interfere with DNA polymerases during replication, introducing base mismatches. Moreover, extreme conditions impair the very systems that would normally correct such errors. Core repair pathways—including base-excision repair, nucleotide-excision repair, mismatch repair, and homologous recombination—rely on precisely structured enzyme complexes and coordinated cascade reactions [106,107,108,109,110,111]. Stressors interfere with these systems at multiple levels: temperature can denature repair enzymes, reduce their substrate-binding affinity, or slow the kinetics of enzymatic reactions [10,112]; and ATP depletion, cofactor scarcity, or redox imbalance induced by stress directly limit repair efficiency [113,114]. When damage outpaces repair, the resulting stall triggers a global stress response—most notably, the prokaryotic SOS system. The specific mutational signatures generated are a direct consequence of the particular DNA damage landscape and the error-prone polymerases recruited. In many bacteria, the SOS response is orchestrated by RecA-mediated cleavage of the LexA repressor. This derepresses Pol V, which generates transversion mutations, particularly AT → TA changes, while Pol IV primarily causes-1 frameshift mutations at homopolymeric sequence motifs. The predominance of one polymerase over another is stress-specific: UV radiation strongly induces Pol V, whereas nutrient starvation or certain alkylating agents may favor Pol IV [115]. This mechanistic specificity means the evolutionary potential is biased by the stressor itself. Whether stress-induced mutagenesis represents an evolved adaptive strategy or an unavoidable byproduct of overwhelmed repair systems remains debated. The bet-hedging interpretation posits that elevated mutation rates are selectively favored in fluctuating environments, where generating phenotypic diversity during rare favorable windows outweighs the cost of deleterious mutations. Alternative models, however, emphasize that the SOS response is fundamentally a damage-tolerance mechanism, with mutagenesis arising as a molecular side effect that selection has not eliminated simply because the benefit of lesion bypass outweighs the fidelity cost. The observation that specific polymerases are preferentially induced by distinct stressor types—Pol V by UV damage, DinB by alkylation stress—lends weight to the adaptive view, suggesting tailored mutagenic responses rather than generic error-prone repair [116,117].

Beyond the direct damage caused by ROS, the cellular redox environment itself modulates mutation rates. A shift toward a more oxidizing intracellular milieu—reflected in altered ratios of reduced to oxidized glutathione (GSH/GSSG) or thioredoxin (Trx(SH)_2_/Trx-S_2_)—can directly impact the activity of DNA repair enzymes, many of which contain redox-sensitive cysteine residues in their active sites [118]. For instance, the DNA glycosylase MutY, which excises misincorporated adenines opposite 8-oxoguanine, is inactivated under oxidizing conditions [119], allowing oxidative lesions to persist into subsequent rounds of replication [120]. Consequently, the elevated mutation rates observed under combined extreme stresses are not solely attributable to increased DNA damage load but also to a stress-imposed relaxation of repair fidelity mediated by redox imbalance [121]. This redox–mutagenesis axis is particularly pronounced in fluctuating extreme environments, where episodic oxidative bursts and reductive recovery cycles alternately impair and restore repair capacity, creating temporally defined windows of hypermutability.

The tempo and mode of this mutation-rate elevation, however, differ fundamentally between the stable and fluctuating extreme environments. In stable extremes, selection is continuous and unidirectional, favoring a sustained but controlled increase in mutagenesis. In fluctuating extremes, pulses of hypermutability during stress episodes are followed by purifying selection during benign intervals, creating a “stop-and-go” evolutionary dynamic. This pulsed pattern of mutagenesis is best understood not as a failure of repair but as an evolved bet-hedging strategy. By rapidly generating genetic variation precisely when survival is threatened, populations increase the probability that at least some individuals will carry adaptive mutations should conditions worsen further. However, to mitigate the inevitable cost of deleterious mutations, microorganisms tightly regulate these pathways, activating them primarily when the survival imperative outweighs the risk of genetic load [122,123,124]. Thus, stress controllably elevates mutation rates, providing a direct mechanistic link between environmental pressure and accelerated genomic evolution.

Beyond direct mutagenesis, stress can also accelerate phenotypic evolution through non-genetic mechanisms. The molecular chaperone Hsp90 normally buffers the phenotypic effects of cryptic mutations by ensuring proper folding of metastable proteins [125]. Under extreme conditions—whether thermal, osmotic, or oxidative—the demand for Hsp90 shifts dramatically toward managing acute protein damage, titrating its activity away from these otherwise buffered clients [126,127]. When this buffering capacity is released, the accumulated genetic variation is suddenly unmasked, revealing novel phenotypes upon which selection can act. This mechanism is particularly consequential in fluctuating extreme environments, where repeated cycles of Hsp90 titration during stress episodes and Hsp90 restoration during benign intervals can sequentially expose and then stabilize cryptic variation, effectively accelerating the phenotypic exploration of sequence space without requiring new mutations [126,127]. This provides a rapid, stress-induced pathway to phenotypic novelty that operates in parallel with elevated mutation rates, and one that directly links environmental fluctuation to the tempo of evolutionary innovation.

Direct evidence that extreme environments accelerate mutation accumulation has emerged from long-term evolution experiments with thermophilic bacteria. Long-term mutation accumulation experiments in thermophilic bacteria have demonstrated elevated rates of hydrolytic DNA damage at high temperatures, with cytosine deamination generating characteristic GC → AT transitions. Critically, mutations affecting DNA repair genes can create a positive-feedback loop in which thermally compromised repair further elevates the mutation rate [128].

### 3.2. Stress-Mediated Enhancement of Horizontal Gene Transfer

Extreme environmental stress enhances the intercellular exchange of genetic material by both weakening physical barriers and actively inducing the molecular machinery of uptake [129,130,131,132]. Stress conditions directly alter membrane lipid composition and fluidity, increasing permeability to extracellular DNA [133,134,135,136,137]. Simultaneously, stress-induced cellular damage or lysis releases substantial genomic DNA into the environment, providing a continuous source of transforming material [138,139,140].

However, increased membrane permeability alone does not fully account for the efficiency of HGT observed in extreme environments. In many bacteria, DNA uptake is not a passive leak but an actively regulated physiological state termed competence. In model organisms such as *Bacillus subtilis*, competence is governed by the ComABCDE quorum-sensing system and the master transcription factor ComK [141]. Stressors such as DNA damage or nutrient limitation can directly induce ComK expression, thereby linking environmental adversity to genetic receptivity [142]. Redox balance plays a similarly underappreciated role in modulating HGT frequency. In several bacterial pathogens, the activation of competence and the induction of prophages are both controlled by the redox-sensitive transcriptional regulator OxyR or by the SOS regulon, which itself responds to the accumulation of single-stranded DNA—a lesion exacerbated under oxidizing conditions that impair replication fidelity [143]. More directly, the formation of the relaxosome during conjugation requires the precise folding of TraI and TraY at the oriT sequence; oxidative modification of the cysteine residues coordinating the relaxase active site can either activate or inhibit cleavage activity depending on the specific redox chemistry of the enzyme [144]. In fluctuating extreme environments, the redox-mediated competence window is likely rhythmically gated, opening during the recovery phase when ROS levels subside and cellular energy charge is restored—precisely when DNA repair pathways are also most active and the integration of newly acquired sequences into the genome is most favorable [145]. This redox gating of HGT adds another layer of temporal structuring to the genetic exchange dynamics in extreme environments. The structure of the uptake machinery itself, specifically the Type IV pilus-like pseudopilus and the ComEC/DNA uptake channel, is evolutionarily tuned for function under specific conditions. For instance, homologous systems in thermophilic bacteria possess thermostable ComEC variants with altered transmembrane domains that maintain channel integrity under the very conditions that increase membrane fluidity [146]. Opinions diverge on whether the elevated HGT frequencies observed in extreme environments reflect active selection for competence or are merely passive consequences of membrane damage and lysis [147,148]. Evidence from these thermophilic competence systems, where ComEC channels and associated pseudopilins display clear thermoadaptive modifications in their transmembrane domains, argues for active maintenance of DNA uptake machinery under persistent thermal stress. However, the contribution of stress-induced membrane leakiness cannot be entirely dismissed, particularly in hyperosmotic environments where lipid remodeling may transiently increase permeability to extracellular DNA.

Beyond competence, stress promotes HGT through additional routes. Conjugation machinery encoded on plasmids can be directly activated by environmental stressors, promoting the lateral dissemination of mobile genetic elements [149,150,151,152]. For conjugation, the relaxosome, composed of the relaxase TraI, the accessory protein TraY, and the origin-of-transfer *oriT* sequence, is essential for initiating DNA transfer; stress-induced DNA damage can generate free linear DNA that provides a substrate for relaxosome activity, accelerating plasmid mobilization [153]. Transduction, mediated by bacteriophages, may further contribute to HGT, particularly under conditions that induce prophage activation in lysogenized host genomes, though its quantitative importance in extreme environments remains less explored relative to transformation and conjugation.

The population-genetic impact of HGT is magnified by intense selection. In stable extreme environments, low population density and stable niche boundaries may limit HGT frequency, favoring vertical inheritance. In contrast, fluctuating extreme environments, with their disturbance-driven DNA release, community mixing, and stress-induced competence, act as hotspots for HGT, where acquired adaptive gene clusters can rapidly sweep through populations.

### 3.3. Spatial Propagation of Stress-Induced Genetic Resources

Beyond driving in situ evolution, extreme environments function as engines of genetic innovation that can influence microbial evolution across much broader spatial scales [154]. Physical vectors such as water flow, aerosolization, or animal vectors can transport viable extremophiles or their free DNA into adjacent non-extreme environments [132,155,156,157]. Upon arrival in these relaxed selective settings, this influx of “foreign” genetic resources can fundamentally alter the evolutionary trajectory of resident mesophilic communities. For example, thermophiles washed out from hot springs into cold streams, while unable to outcompete native populations, may serve as vectors for stress-tolerance genes via HGT [132,158]. This “gene spillover” effect implies that the evolutionary impact of an extreme environment extends far beyond its physical boundaries, creating a “genetic subsidy” for surrounding ecosystems. Stable extreme environments may produce rare but impactful genetic exports. Fluctuating extreme environments, as dynamic engines of innovation, likely serve as more consistent and prolific sources of pre-assembled adaptive modules for the broader biosphere.

## 4. Stress-Driven Reorganization of Ecological Networks

### 4.1. Genetic Novelty Rewires Interaction Edges

**Figure 2:**
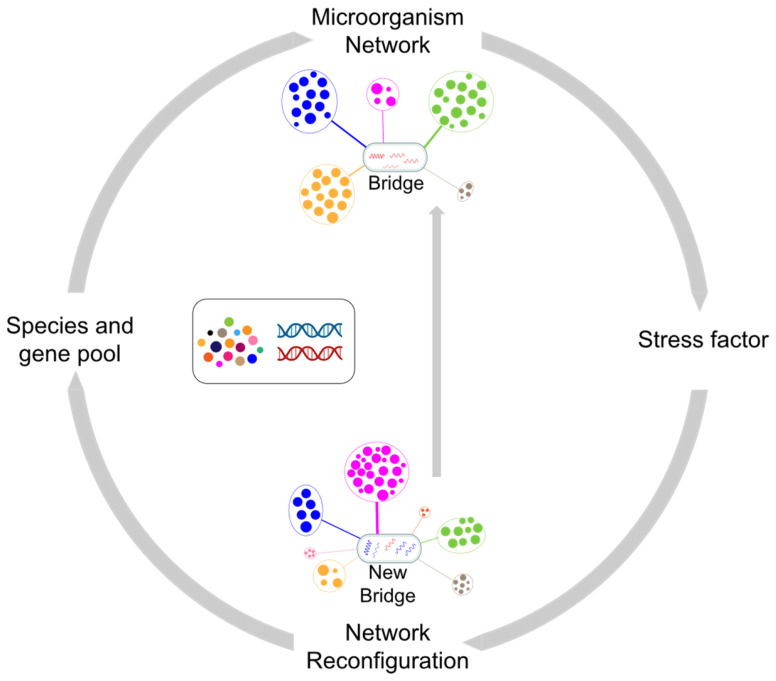
Stress-driven adaptive reconfiguration of microbial ecological networks. Different colored circles represent distinct microbial species, with circle size proportional to population size. Red and blue DNA indicate genetic material of different origins. Different colored solid lines of varying weight denote different types and strengths of interspecies interactions. Stress factors alter network topology by shifting species abundance, connectivity, and centrality, producing a reconfigured network with new hubs (New Bridge) and altered interaction architecture.

Persistent stress in extreme environments, by altering the rules of species interactions, systematically drives the structural and functional reorganization of entire community ecological networks. Gene acquisitions, losses, or functional innovations at the micro-scale first alter the niche of a population, redefining the nature and strength of its interactions with other community members, leading to the creation, loss, or re-weighting of links—the rewiring of specific interaction edges—which ultimately drives fundamental changes in global network properties (Figure 2).

The acquisition of novel functions is a key driver for creating new network hubs and expanding connectivity [159,160,161]. For instance, if a previously peripheral species acquires, via HGT, a key set of enzymes for degrading a toxic pollutant such as polycyclic aromatic hydrocarbons, it transforms from an ordinary heterotroph into the primary agent for breaking down that compound [162,163]. This functional evolution immediately expands its niche, attracting a range of follower species that depend on its degradation products, thereby forming a dense, cooperative module centered on this detoxifier [164,165]. Topologically, this manifests as a sharp increase in the connectedness and bridging role of this new central player. Conversely, the loss of critical cooperative functions can trigger module disintegration and network simplification [159,161,166]. Furthermore, the evolution of competitive and defensive mechanisms directly reshapes interaction patterns and network stability. For instance, acquiring aggressive traits like a type VI secretion system introduces strong antagonistic interactions into the network [167,168], often driving the network toward a sparser, more antagonistic, and segregated structure.

### 4.2. The Rise and Fall of Modules and Hubs

The changes in individual interaction edges accumulate to drive the reconfiguration of global network architecture—measurable shifts in topological properties such as modularity, centrality distribution, and connection density—which directly regulates functional output and stability. The shifts in modularity are directly linked to the community function and robustness [169,170]. A highly modular network implies the formation of several tightly connected, functionally semi-autonomous formations within the community [171,172]. This structure provides a buffering effect: disturbance affecting non-core species within one module may be contained locally without cascading into system-wide collapse. However, if the lost species is a keystone bridge-node connecting different modules, the metabolic coupling or signalling between modules will be disrupted [159,173]. Shifts in centrality serve as topological markers of the dynamic relocation of functional cores. The identity of these bridge-nodes—and thus the vulnerability of the entire network—depends heavily on which environmental parameter dominates community organization. Among the strongest organizers is redox potential.

A particularly powerful illustration of stress-driven network reorganization is observed along redox gradients. In deep-sea hydrothermal vents or stratified saline lakes, the spatial transition from oxidized to reduced fluids dictates a cascade of electron acceptors: O_2_ → NO_3_^−^ → Mn(IV)/Fe(III) → SO_4_^2−^ → CO_2_. This abiotic gradient serves as a hard template for community assembly. The transition from sulfate-reducing to methanogenic zones represents a clear network boundary where bridge species—such as syntrophic bacteria capable of anaerobic methane oxidation (ANME)—emerge as the sole topological links [174]. The failure of these specific bridge nodes, which operate under extreme thermodynamic constraints, can lead to cascading module collapse far more abruptly than in oxic zones [175]. This underscores how the type of stress imposes specific, predictable architectural features on the interaction network. The molecular underpinnings of such topology-defining interactions are increasingly accessible. In the sulfate-methane transition zones of deep-sea sediments, the syntrophic partnership between anaerobic methanotrophic (ANME) archaea and sulfate-reducing bacteria is mediated by direct interspecies electron transfer (DIET) via multi-heme cytochromes and, in some consortia, conductive pili [176]. Disruption of the genes encoding these electron-transfer structures does not merely impair metabolism; it severs the sole topological link between the methane-oxidizing and sulfate-reducing modules, causing the network to fragment into two disconnected subgraphs. This experimentally confirms that the bridge-node status of these syntrophic partners is not merely a network inference artifact but is physically instantiated in specific, identifiable gene products.

This principle extends beyond redox chemistry to the broader contrast between stable and fluctuating extreme environments. Stable extreme environments tend to produce networks with high modularity but sparse inter-module connections and stable hubs, reflecting predictable selection. Fluctuating extreme environments, conversely, favor high modularity coupled with more numerous and dynamic bridge species, reflecting the need to buffer and adapt to shifting stress regimes. In this light, the redox-stratified network represents an extreme case of the stable-environment configuration: its bridge-nodes are chemically defined by thermodynamic thresholds, and their failure modes are correspondingly abrupt. The redox architecture of these networks, by defining which species serve as bridge-nodes, also indirectly channels the evolutionary trajectory of the community: the intense selective pressure concentrated on these thermodynamically constrained hubs favors the acquisition—via HGT—of metabolic modules that expand the electron acceptor repertoire, thereby feeding back into the network topology by potentially creating new interaction links.

### 4.3. The Network as an Evolutionary Filter

The newly formed network is not an evolutionary endpoint. It reflexively becomes a powerful and structured selective environment that guides subsequent micro-evolution, creating a feedback loop. This entire process—from stress-triggered rewiring through topological reconfiguration to the establishment of a new stable architecture—constitutes ecological network reorganization.

Specifically, when a tolerant species rises to become a central hub, the dense connections it forms with its syntrophic partners create a highly interdependent local module [177,178]. For this hub species, subsequent selection will strongly favor maintaining and optimizing its core service function; any mutation that weakens this function will be under strong negative selection [179]. Meanwhile, for its dependent species, selection will favor traits that enhance the efficiency of utilizing this specific resource [180,181,182,183]. This “interaction-mediated selection” constrains the evolutionary paths according to network roles. This completes the dynamic, layered feedback loop: initial stress triggers genetic innovation and network reorganization; the restructured network then defines new selective rules based on topology and ecological interactions; and subsequent evolution proceeds under these new rules, further consolidating the network architecture. This loop underscores that a community’s evolutionary trajectory is not dictated solely by the external environment but is profoundly shaped by its own evolving internal structure.

### 4.4. Resilience Through Adaptive Restructuring

The ultimate consequence of this feedback cycle is enhanced system resilience [184]. In extreme environments, system resilience refers to the capacity to absorb disturbances while maintaining core structure, functions, and key ecological processes essentially unchanged [185,186,187].

Mechanistically, system resilience is rooted in multi-layered buffering capacities. At the abiotic-stress level, communities can mitigate stress through collective responses, such as the cooperative synthesis of compatible solutes [188,189]. At the network topology level, a community with high modularity confines damage to specific modules, preventing cascading collapse [190], while functional redundancy allows the network to redistribute flux and preserve overall functional output [188,191,192].

The most profound manifestation of system resilience under extreme conditions, however, lies in its capacity for adaptive transformation through evolutionary innovation. When disturbance intensity exceeds resistance and recovery thresholds, the system may initiate a process of adaptive restructuring, including HGT, rapid evolution, and topological restructuring [193,194,195,196]—enabling it to convert a formerly harmful compound into a resource or establish a new stable state [197,198].

Therefore, resilience in extreme environments is not merely resistance to change, but the capacity for stress-induced adaptive reorganization. It is sustained by the continuous feedback between accelerated evolution and network reorganization.

## 5. Convergent Evidence from Natural Systems and Experimental Evolution

The stress-driven acceleration of microbial evolution and its ecological consequences are supported by a convergent, multi-tiered body of evidence. This evidence spans macro-temporal patterns in Earth’s history, genomic-level mechanisms observed in natural populations, and direct experimental validation under controlled laboratory conditions. Together, they construct a coherent chain linking intense environmental selection to rapid genomic innovation, and subsequently to the functional reorganization of ecological networks.

On geological timescales, the evolutionary trajectories of extremophilic lineages are tightly coupled with periods of major environmental upheaval, such as Korarchaeota and the Great Oxidation Event (~2.42 billion years ago) [199]. This indicates that global extremes acted as intense selective drivers, compressing the time required for major adaptive radiations. Such deep-time records are more likely to capture the stable, unidirectional evolutionary outputs of stable extreme environments. The rapid, oscillating dynamics of fluctuating extreme environments, in contrast, may leave subtler, higher-frequency signatures in the paleontological and genomic record.

At the genomic level, accelerated evolution is evidenced by the rapid acquisition and remodeling of genetic material. Pangenome analyses of poly-extremotolerant bacteria like *Exiguobacterium* show that distinct ecotypes arise through the gain or loss of specific metabolic gene clusters, while obligate symbionts demonstrate efficient, selection-driven genome streamlining [200,201].

While genomic signatures from natural populations document the end-products of stress-driven evolution, Adaptive Laboratory Evolution (ALE) provides direct, causal validation of the underlying dynamics [202]. By imposing defined, intensifying stressors, ALE forces microbial populations to accumulate adaptive mutations over hundreds to thousands of generations [203]. It is crucial to note that standard ALE experiments, which impose constant selective pressure, most closely model the evolutionary dynamics of stable extreme environments. To simulate fluctuating dynamics, future experiments should employ oscillating stress regimes, periodically switching stressor type or intensity [204,205]. This distinction is essential for using ALE to probe the full spectrum of stress-driven evolution.

## 6. Conclusions

This review has argued that persistent abiotic stress in extreme environments drives a self-reinforcing feedback loop. Accelerated genomic innovation—via elevated mutation rates, enhanced horizontal gene transfer, and chaperone-mediated release of cryptic variation—continuously supplies new traits that reorganize ecological network architecture. The restructured network, in turn, generates novel selective pressures that channel subsequent evolution. The outcome of this reciprocal dynamic is not passive resistance but a distinctive capacity for adaptive reorganization.

Grounding this framework more firmly requires recognizing two points. First, extremophile adaptation is not the elimination of stress but its energy-intensive, continuous management. Second, environments impose qualitatively different evolutionary regimes depending on whether their stressors are stable or fluctuating; the latter serve as particularly potent engines of genetic and topological turnover.

It must also be acknowledged that these extreme systems function not in isolation but as sources of genetic subsidy to the surrounding biosphere through gene spillover. Yet substantial gaps persist. The molecular regulation of mutagenic and horizontal transfer pathways has been dissected largely in model mesophiles rather than in phylogenetically deep-branching extremophiles. Network-level inferences remain predominantly correlative, and the temporal mismatch between evolutionary dynamics and experimental observation limits causal certainty [206,207].

Addressing these gaps calls for time-resolved meta-omics, adaptive laboratory evolution under oscillating stress regimes, and the construction of defined synthetic consortia for controlled network rewiring experiments. Within this landscape, three questions are now tractable: whether network modularity scales with the variance rather than the mean of abiotic stress, whether constitutive chaperone investment imposes a measurable trade-off against metabolic evolvability, and whether synthetic communities under sustained stress recapitulate the bridge-node turnover observed in natural systems. Beyond their fundamental interest, the principles emerging from this feedback loop offer a blueprint for evolutionary engineering, where the deliberate manipulation of stress regimes—pulsed versus continuous—can direct microbial consortia toward functional stability or novel catabolic pathways for applications in bioremediation, bioprocessing, and ecosystem management [208].

## Figures and Tables

**Table 1:** Primary impacts of extreme environmental stresses on microorganisms.

Stress Type	Primary Mechanism	Key Targets & Direct Effects
High Temperature	Macromolecular denaturation and membrane hyperfluidization	DNA/RNA: strand separation, increased hydrolytic damage, replication interference Proteins: unfolding, aggregation, irreversible denaturation Membranes: increased fluidity and permeability, disrupted ion gradients Metabolism: ATP depletion from futile cycles and repair costs Cell integrity: impaired division, heat-shock regulon activation
Low Temperature	Phase transitions, ice crystallization, metabolic suppression	DNA/RNA: stabilized secondary structures impeding replication and transcription Proteins: cold denaturation, reduced enzymatic activity, cold-shock protein induction Membranes: gel-phase transition, loss of permeability control, impaired transport Metabolism: suppressed enzyme kinetics, metabolic quiescence Cell integrity: ice crystal damage, freeze–thaw injury, osmotic shock
Hyperosmotic stress	Low water activity and ionic imbalance	DNA/RNA: increased supercoiling, altered gene expression Proteins: altered solvation and conformation, osmoprotectant synthesis Membranes: reduced fluidity, altered curvature, impaired transporter function Metabolism: high-ATP-cost compatible solute accumulation Cell integrity: cytoplasmic shrinkage, loss of turgor, impaired division
High Pressure	Compression of biomolecular volume and altered reaction equilibria	DNA/RNA: stabilized helix, altered supercoiling, helicase/polymerase interference Proteins: denaturation, oligomer dissociation, altered binding equilibria Membranes: tighter lipid packing, reduced fluidity, phase transitions Metabolism: altered reaction kinetics from activation volume effects Cell integrity: impaired motility and cytokinesis
Desiccation	Removal of hydration shell and extreme low water activity	DNA/RNA: strand breaks, abasic sites, Maillard crosslinks Proteins: loss of activity, irreversible aggregation, Maillard adducts Membranes: non-lamellar phase transitions, vesiculation, rehydration-induced disruption Metabolism: metabolic dormancy, ATP depletion Cell integrity: cytoplasmic vitrification, osmotic shock during rehydration
Extreme pH	Disruption of charged residues and ion gradients	DNA/RNA: acid depurination, alkaline hydrolysis, altered DNA-protein interactions Proteins: altered charge, disrupted salt bridges, misfolding, aggregation Membranes: compromised integrity, altered permeability Metabolism: dissipated proton motive force, increased ATP demand for ion extrusion Cell integrity: loss of cytoplasmic pH homeostasis, impaired nutrient transport
Radiation	Direct energy absorption and indirect ROS generation	DNA: single- and double-strand breaks, pyrimidine dimers, oxidative base modifications Proteins: backbone cleavage, cysteine/methionine oxidation, carbonylation Membranes: lipid peroxidation, loss of fluidity and integrity Metabolism: depletion of antioxidant pools, disrupted electron transport Signaling: SOS response activation, cell cycle arrest, filamentation
Oxidative Stress	ROS accumulation and thiol-disulfide redox imbalance	DNA: oxidative base modifications, strand breaks Proteins: cysteine/methionine oxidation, carbonylation, disulfide scrambling, repair enzyme inactivation Membranes: peroxidation of unsaturated fatty acids, increased permeability Metabolism: NADPH depletion, thioredoxin/glutaredoxin pool exhaustion, Fe-S cluster disruption Signaling: OxyR/SoxRS/PerR regulon activation, indirect mutation rate elevation via repair fidelity loss
Reductive Stress	Excess of reducing equivalents (elevated NADH/NAD^+^ and NADPH/NADP^+^ ratios)	DNA: imbalanced dNTP pools, replication fork progression interference Proteins: reductive unfolding, inappropriate disulfide bond reduction Membranes: altered lipid composition from excess NADPH Metabolism: inhibited catabolic dehydrogenases, overflow metabolism, impaired electron transfer Redox homeostasis: over-reduced ferredoxin/thioredoxin pools, FTR system engagement, transcriptional reprogramming via NADH-responsive regulators

## Data Availability

This review does not involve the creation of new data.
